# Gut Microbiota-Dependent Trimethylamine *N*-Oxide Pathway Associated with Cardiovascular Risk in Children with Early-Stage Chronic Kidney Disease

**DOI:** 10.3390/ijms19123699

**Published:** 2018-11-22

**Authors:** Chien-Ning Hsu, Pei-Chen Lu, Mao-Hung Lo, I-Chun Lin, Guo-Ping Chang-Chien, Sufan Lin, You-Lin Tain

**Affiliations:** 1 Department of Pharmacy, Kaohsiung Chang Gung Memorial Hospital and College of Medicine, Chang Gung University, Kaohsiung 833, Taiwan; chien_ning_hsu@hotmail.com; 2 School of Pharmacy, Kaohsiung Medical University, Kaohsiung 807, Taiwan; 3 Department of Pediatrics, Kaohsiung Chang Gung Memorial Hospital and College of Medicine, Chang Gung University, Kaohsiung, 833, Taiwan; alexiellu@gmail.com (P.-C.L.); trentlo@cgmh.org.tw (M.-H.L.); uc22@cgmh.org.tw (I.-C.L.); 4 Center for Environmental Toxin and Emerging-Contaminant Research, Cheng Shiu University, Kaohsiung 833, Taiwan; guoping@csu.edu.tw (G.-P.C.-C.); linsufan2003@gmail.com (S.L.); 5 Super Micro Mass Research and Technology Center, Cheng Shiu University, Kaohsiung 833, Taiwan

**Keywords:** ambulatory blood-pressure monitoring, arterial stiffness, cardiovascular disease, children, chronic kidney disease, gut microbiota, hypertension, trimethylamine *N*-oxide, pulse-wave velocity

## Abstract

Despite cardiovascular disease (CVD) being the leading cause of morbidity and mortality in chronic kidney disease (CKD), less attention has been paid to subclinical CVD in children and adolescents with early CKD stages. Gut microbiota and their metabolite, trimethylamine *N*-oxide (TMAO), have been linked to CVD. Ambulatory blood-pressure monitoring (ABPM) and arterial-stiffness assessment allow for early detection of subclinical CVD. We therefore investigated whether gut microbial composition and TMAO metabolic pathway are correlated with blood-pressure (BP) load and vascular abnormalities in children with early-stage CKD. We enrolled 86 children with G1–G3 CKD stages. Approximately two-thirds of CKD children had BP abnormalities on ABPM. Children with CKD stage G2–G3 had a higher uric acid level (6.6 vs. 4.8 mg/dL, *p* < 0.05) and pulse-wave velocity (4.1 vs. 3.8 m/s, *p* < 0.05), but lower TMAO urinary level (209 vs. 344 ng/mg creatinine, *p* < 0.05) than those with stage G1. Urinary TMAO level was correlated with the abundances of genera *Bifidobacterium* (*r* = 0.307, *p* = 0.004) and *Lactobacillus* (*r* = 0.428, *p* < 0.001). CKD children with abnormal ABPM profile had a lower abundance of the *Prevotella* genus than those with normal ABPM (*p* < 0.05). Our results highlight the link between gut microbiota, microbial metabolite TMAO, BP load, and arterial-stiffness indices in children with early-stage CKD. Early assessments of these surrogate markers should aid in decreasing cardiovascular risk in childhood CKD.

## 1. Introduction

Children and adolescents with chronic kidney disease (CKD) are at high risk of developing cardiovascular disease (CVD) [1]. Unlike adults, overt CVD rarely presents during childhood. Thus, surrogate markers have been required to manage and stratify risk for CVD in youths. Surrogates may be biomarkers, structural markers, or functional markers. Examples of structural and functional markers are the noninvasive measurement of blood-pressure (BP) load, arterial stiffness, and vascular phenotype [2]. We and others have shown that more than half of children and adolescents with mild-to-moderate CKD develop abnormal BP patterns on 24 h ambulatory blood-pressure monitoring (ABPM) [3,4]. However, variations of carotid artery intima–media thickness (cIMT), an assessment of vascular phenotype, in different stages of pediatric CKD observed can be elevated [5] or unaltered [6]. Additionally, arterial-stiffness indices, such as pulse-wave velocity (PWV) and ABPM-derived arterial-stiffness index (AASI), have been linked to known risk factors of CVD in adult CKD patients [7]. Although high PWV and CKD have been reported as predictors of CV events in adult patients [8], it remains unclear whether various arterial-stiffness indices are related to cardiovascular risk in children with early-stage CKD.

Recent studies suggest a pathogenic association between gut microbiota and CKD [9,10]. CKD can affect microbial composition, leading to gut dysbiosis, while gut dysbiosis in CKD patients may increase gut microbiota-derived uremic toxins that, in turn, contribute to CKD progression [9,10]. Although some microbial markers have been linked to CVD and hypertension [11,12], the impact of gut microbiota on the BP load and its association with structural and functional surrogate markers in children with CKD have not been studied.

Trimethylamine *N*-oxide (TMAO), a gut microbiota-derived metabolite, is considered a uremic toxin [13]. Dietary choline and carnitine are metabolized by gut microbes to generate trimethylamine (TMA), which, in turn, is metabolized by flavin monooxygenases (FMOs) to form TMAO. Recent studies have demonstrated an association between blood TMAO levels and the risk of CKD, CVD, atherosclerosis, nonalcoholic fatty liver disease, and metabolic syndrome [14,15,16,17]. However, less attention has been paid to the study of urinary TMAO level in these diseases. The relationship between urine TMAO level, alterations of gut microbiota, and CVD risk in children with early-stage CKD especially remains largely unknown. Although TMAO is mainly produced from TMA by FMO, TMAO and TMA can be converted to dimethylamine (DMA). Thus, simultaneous measuring of TMA, TMAO, and DMA may provide us with a whole picture of the TMA–TMAO metabolic pathway involved in CVD in CKD children. Since blood TMAO level has been well-studied in CKD [15,17], in the current study we therefore mainly focused on urinary TMAO and its metabolites. Because the urinary-excretion mechanism is required to counteract the accumulation of blood TMAO, we hypothesized that CKD severity would affect the urinary excretion of TMAO-related metabolites, and their levels in the urine could be markers for CVD in children with early-stage CKD.

Therefore, the aim of this study was to identify the associations between gut microbial composition, the TMA–TMAO metabolic pathway, BP load, and arterial-stiffness indices in children and adolescents with early-stage CKD.

## 2. Materials and Methods

### 2.1. Study Population

The prospective cohort study was approved by the Institution Review Board and Ethics Committee of Chang Gung Medical Foundation, Taoyuan, Taiwan (Permit number: 201601181A3; approval date: 30/12/2016) and adherent to the principle of 1964 Declaration of Helsinki and its later amendments. Informed consent was obtained from all participants before the study. From December 2016 through September 2018, children aged 3 to 18 years with CKD from the pediatric nephrology clinics in a Taiwan medical center were recruited. CKD refers to kidney damage or decreased kidney function of over at least three months [18]. Kidney damage is defined as structural or functional abnormalities, established via renal biopsy or imaging studies, or inferred from markers, such as urinary-sediment abnormalities or proteinuria. Renal function was determined by estimated glomerular filtration rate (eGFR) using the Schwartz formula according to body height and serum-creatinine (Cr) levels [19]. Patients were excluded if they (1) were an already documented pregnancy; (2) had a history of congenital heart disease; (3) had eGFR < 15 mL/min/1.73 m^2^, were on dialysis maintenance, or ever received renal transplantation; or (4) were unable to follow up protocol or co-operate with assessment. All participants were categorized to eGFR category G1 (eGFR ≥ 90 mL/min/1.73 m^2^), G2 (eGFR 60–89 mL/min/1.73 m^2^), or G3 (eGFR 30–59 mL/min/1.73 m^2^). Analysis was restricted to children with a baseline eGFR >15 mL/min/1.73m^2^; measured renal and cardiovascular parameters are described in the following section. In the current study, we enrolled a total of 86 children and adolescents with CKD stage G1 to G3. The causes of kidney diseases were divided to two categories: Congenital anomalies of the kidney and urinary tract (CAKUT), or non-CAKUT. CAKUT structural anomalies range from renal agenesis, kidney hypo-/dysplasia, multicystic kidney dysplasia, horseshoe kidney, duplex collecting system, posterior urethral valves, and ureter abnormalities [20].

### 2.2. Biochemical Analysis

Fasting plasma specimens, spot urine, and fecal samples were aliquoted and stored at –80 °C until analysis. Blood urea nitrogen, creatinine, uric acid, glucose, total cholesterol, LDL, triglyceride, sodium, potassium, calcium, phosphate, hemoglobin, and urine total protein-to-creatinine ratio were measured by hospital central laboratory. We directed the family to have their children avoid excessive intake of foods rich in choline and carnitine (e.g., eggs, fish, or red meat) for 1 week before blood and urine sampling.

### 2.3. Office BP and 24-Hour ABPM

Three BP readings were obtained by trained specialist nurses using a standard mercury sphygmomanometer (cuff with bladder size by a 1:2 width to length ratio according to arm circumference). BP measurements were taken at clinic visit after 5 min sitting rest with at least 1 min between recordings. The mean value was used as the participant’s office BP for analysis. The 24 h ABPM data were collected for subjects aged 6–18 years using an Oscar II monitoring device (SunTech Medical, Morrisville, NC), handled by an experienced specialist nurse as previously reported [6]. Briefly, the ABPM was set to record the BP and pulse rate at 20 min intervals over 24 h. The subjects and their parents were asked to keep a diary of sleeping and waking times, as well as activities that may influence BP measurements, including exercise and stressful situations. Only measurements with a systolic BP of 50–200 mm Hg, a diastolic BP of 30–100 mm Hg, and a heart rate of 30–200 beats per minute were accepted as valid and included in analysis. If more than 25% of an individual’s recordings were outside these valid ranges, then that individual was excluded from further analysis.

An abnormal ABPM profile was determined based on (1) awake, asleep, systolic, or diastolic BP loads exceeding the 95th percentile based on gender and height using ABPM reference data [21]; (2) awake, asleep, systolic or diastolic BP load of 25% or greater; and (3) asleep decrease of BP load by less than 10% compared with average awake BP load. Next, diastolic BP was plotted against systolic BP using the individual 24 h ABPM readings to calculate the linear-regression slope. The AASI was defined as 1 minus the regression slope [22]. 

### 2.4. Carotid Ultrasonography

The carotid ultrasound studies were performed by two experienced pediatricians (P.-C.L. and I.-C.L.) as previously reported [6]. Patients was in the supine position for at least 10 min in a quiet room prior to examination. With their neck hyperextended and turned 30–45 degrees contralaterally to the probe, the bilateral mid common carotid artery was imaged using a 5 to 12 MHz linear array transducer. We used the distance between the leading edges of the luminal–intimal interface and the medial–adventitial interface for the measurement of cIMT. The cIMT was measured during end diastole as determined by the R wave on the electrocardiogram. These images were obtained with the ProSound α7 ultrasound coupled to computer-assisted analysis software (e-TRACKING system; Aloka Co., Tokyo, Japan). Arterial-stiffness index PWV was determined by echo-tracking methods (e-TRACKING system; Aloka Co., Tokyo, Japan).

### 2.5. Liquid Chromatography–Mass Spectrometry (LC–MS) Analysis

Urinary DMA, TMA, and TMAO levels were measured by LC–MS analysis using an Agilent 6410 Series Triple Quadrupole mass spectrometer (Agilent Technologies, Wilmington, DE, USA) equipped with an electrospray ionization source. DMA, TMA, and TMAO were monitored in multiple-reaction-monitoring mode using characteristic precursor-product ion transitions: m/z 46.1→30, m/z 60.1→44.1, and m/z 76.1→58.1, respectively. An Agilent Technologies 1200 HPLC system was equipped with a binary pump and an autosampler. Chromatographic separation was performed on a SeQuant ZIC-HILIC column (150 × 2.1 mm, 5 μm; Merck KGaA, Darmstadt, Germany) protected by an Ascentis C18 column (2 cm × 4 mm, 5 μm; Merck KGaA, Darmstadt, Germany). The mobile phase containing methanol with 15 mmol/L ammonium formate (phase A) and acetonitrile (phase B) was used at a ratio of 20:80 (phase A: phase B) with a flow rate of 0.3–1 mL/min. Diethylamine was added to plasma samples as an internal standard. The urinary concentration of each metabolite was corrected for urine Cr concentration, which was represented in ng/mg Cr.

### 2.6. Analysis of Gut-Microbiota Composition

Metagenomic DNA was extracted from frozen fecal samples after centrifugation. As described previously [23], all polymerase chain-reaction amplicons were mixed together and sent to the Genomic and Proteomic Core Laboratory, Kaohsiung Chang Gung Memorial Hospital (Kaohsiung, Taiwan) for sequencing using an Illumina Miseq platform (Illumina, CA, USA). Amplicons were prepared according to the 16S Metagenomics Sequencing Library Preparation protocol (Illumina, CA, USA), and sequenced with the Illumina MiSeq platform (Illumina, CA, USA) in paired-end mode with 600-cycle sequencing reagent. Next-generation sequencing data were analyzed with the Microbial Genomics Module of CLC Genomics Workbench 9.5.4 (Qiagen, Stockach, Germany). Taxonomic relative-abundance profiles (e.g., phylum and genus) were compared using the Mann–Whitney U test for independent samples. 

### 2.7. Statistical Analysis

Data were expressed as median and interquartile range (IQR). The Mann–Whitney U test or chi-square test was used to test the differences in variables between children with CKD stage G1 and those with CKD stage G2–G3. The associations between variables were examined using Pearson’s correlation coefficient. A value of *p* < 0.05 was considered statistically significant. Analyses were performed using Statistical Package for the Social Sciences (SPSS) software 14.0 (Chicago, IL, USA).

## 3. Results

We enrolled a total of 86 children and adolescents with CKD stage G1–G3 in this study. Our study population was slightly predominantly male (M:F = 1.7:1), and 64% of participants had a case of CAKUT. As shown in Table 1, children with CKD stage G2–G3 were older, and had higher systolic BP, plasma levels of blood urea nitrogen, creatinine, and uric acid, but lower eGFR compared to those with CKD stage G1.

Among the 86 patients included, 29 cases (34%) were found to exceed the 95th percentile for age, gender, and height by office BP measurements. A total of 54 patients (63%) aged 6–18 years had undergone complete 24 h ABPM studies (Table 2). Among them, 65% (35/54) of children and adolescents with CKD stage G1–G3 had at least one BP load abnormality on 24 h ABPM in this study (Table 2). The ABPM identified 6 (11%), 7 (13%), and 13 patients (24%) with SBP or DBP load > 95th percentile at 24 h, awake, and asleep stages, respectively. Other ABPM abnormalities included 28 patients (52%) with BP load ≥ 25% and 22 patients (41%) with nocturnal SBP nondipping. Except for a BP load ≥ 25%, there was no difference in BP-load abnormalities on ABPM between patients with CKD stage G2–G3 and those with stage G1. The PWV, an arterial-stiffness index, was higher in children with CKD stage G2–G3 compared to those with G1. However, both cIMT and AASI were not different between CKD children with stage G1 and G2–G3.

As shown in Table 3, children with CKD stage G2–G3 had a lower urinary level of DMA and TMAO compared to those with CKD stage G1. However, the urinary levels of DMA, TMA, and TMAO were not different between CKD children with and without BP abnormalities on ABPM. Using data pooled from all subjects (CKD stage G1–G3), correlations of biochemical data, BP load, aortic-stiffness indices, and TMA–TMAO metabolites were analyzed. We observed that blood uric acid level was positively correlated with systolic (*r* = 0.557, *p* < 0.001) and diastolic BPs (*r* = 0.352, *p* < 0.001). Systolic BP was positively correlated with PWV (*r* = 0.605, *p* < 0.001) and urinary DMA level (*r* = –0.281, *p* = 0.009). Both arterial-stiffness indices, PWV (*r* = 0.395, *p* = 0.002) and AASI (*r* = 0.351, *p* = 0.009), were positively correlated with uric acid level. Additionally, PWV was positively correlated with awake SBP load (*r* = 0.491, *p* < 0.001), awake DBP load (*r* = 0.385, *p* = 0.004), asleep SBP load (r = 0.508, *p* < 0.001), and asleep DBP load (*r* = 0.393, *p* = 0.003). It is noteworthy that there were several CV surrogate markers correlated to each other, indicating a close link between uric acid, arterial stiffness, BP load, and the TMA–TMAO pathway.

**Figure 1:**
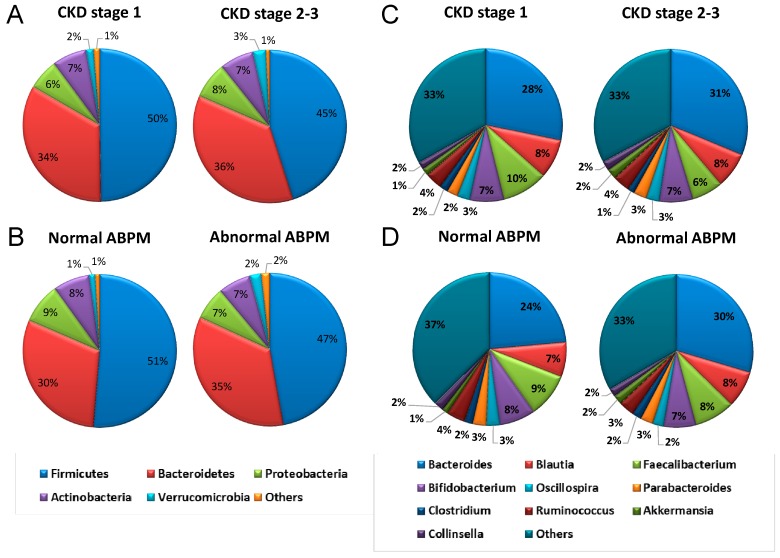
Relative abundances of top five phyla of gut microbiota in children with (**A**) CKD stage G1–G3 and (**B**) normal and ABPM profile. Relative abundances of top 10 genera of the gut microbiota in children with (**C**) CKD stage G1–G3 and (**D**) normal and abnormal ABPM profile.

We further analyzed gut-microbiota composition of children and adolescents with CKD stage G1–G3. We observed that the main phyla in children with CKD were *Firmicutes, Bacteroidetes, Proteobacteria, Actinobacteria*, and *Verrucomicrobia*. At the phylum level, the abundances of main phyla were not different between children with CKD stage G1 and G2–G3 (Figure 1A). The abundances of these main phyla also did not differ between CKD children with a normal and abnormal ABPM profile (Figure 1B). Although the *Firmicutes* to *Bacteroidetes* ratio was reported as a microbial biomarker for hypertension [12], this was not supported by our results in children with early-stage CKD.

**Figure 2:**
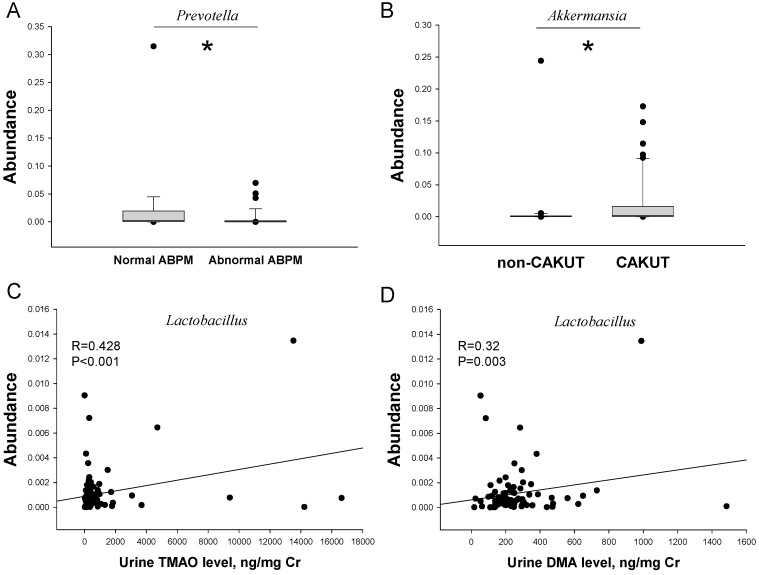
(**A**) Abundance of genus *Prevotella* in CKD children with normal vs. ABPM. (**B**) Abundance of the *Akkermansia* genus in CKD children with CAKUT vs. non-CAKUT. Correlations between abundance of the *Lactobacillus* genus, (**C**) urinary TMAO level, and (**D**) DMA level. * *p* < 0.05 by the Mann–Whitney U-test. The symbol of blackball indicates each recruiting participant.

At the genus level, the top ten bacterial genera were *Bacteroides, Blautia, Faecalibacterium, Bifidobacterium, Oscillospira, Parabacteroides, Clostridium, Ruminococcus, Akkermansia*, and *Collinsella* (Figure 1C). Our results demonstrated that abundances of the main bacterial genera were comparable in CKD children with different stages of CKD (Figure 1C), as well as between normal and abnormal ABPM (Figure 1D). However, CKD children with abnormal ABPM had a lower abundance of the *Prevotella* genus than those with normal ABPM (Figure 2A). Interestingly, children with CAKUT had increased abundance of the genera *Faecalibacterium* (*p* = 0.044), *Bifidobacterium* (*p* = 0.026), and *Akkermansia* (*p* = 0.012) (Figure 2B), but decreased abundance of the *Blautia* genus (*p* = 0.025). Both genera *Bifidobacterium* and *Lactobacillus* have been reported to be the beneficial probiotic bacterial strains [20].

Table 4 lists the correlation between gut microbial composition and other cardiovascular surrogate markers (i.e., arterial-stiffness indices and the TMA–TMAO metabolic pathway). We observed that the abundances of the *Lactobacillus* genus (*r* = –0.284, *p* = 0.029) were negatively correlated with cIMT. PWV and AASI were not correlated with alterations of gut-microbiota components. Additionally, the abundances of genera *Blautia* (*r* = 0.307, *p* = 0.004), *Bifidobacterium* (*r* = 0.36, *p* = 0.001), *Collinsella* (*r* = 0.254, *p* = 0.018), and *Lactobacillus* (*r* = 0.428, *p* < 0.001) (Figure 2C) were positively correlated with the urinary TMAO level. Similarly, urinary DMA level was positively correlated with the abundance of genera *Bifidobacterium* (*r* = 0.227, *p* = 0.036) and *Lactobacillus* (*r* = 0.32, *p* = 0.003) (Figure 2D). However, urinary TMA level was not correlated with alterations of gut-microbiota components.

## 4. Discussion

This study provides insight in a link between gut microbiota and cardiovascular risk in children and adolescents with early-stage CKD. The key findings are as follows: (1) 65% of children and adolescents with CKD stage G1–G3 had BP abnormalities on ABPM; (2) PWV, an arterial-stiffness index, was significantly elevated in children with CKD stage G2–G3 compared to those with stage G1; (3) children with CKD stage G2–G3 had a lower urinary level of DMA and TMAO; (4) CKD children with an abnormal ABPM profile had lower abundance of the genus *Prevotella* than those with normal ABPM; and (5) the abundances of genera *Bifidobacterium* and *Lactobacillus* were correlated with urinary TMAO level.

In line with previous studies in pediatric CKD [3,4,24], we documented a remarkably high prevalence of BP abnormalities on ABPM in children with early-stage CKD. High BP load may develop in the early course of CKD. In the current study, up to 57% of children, even in CKD stage 1, exhibited BP abnormalities. These abnormal ABPM profiles included day- and night-time hypertension, increased BP load, and nocturnal BP nondipping. Our data support the notion that detection of childhood hypertension has significantly improved with the use of ABPM.

In our study population of 86 children with early-stage CKD, hyperuricemia was the only cardiovascular risk factor found to be associated with BP load and arterial-stiffness indices PWV and AASI [25]. Although a recent study showed hyperuricemia-induced hypertension is related to arterial stiffness in adults [26], few data are available examining the relationship between arterial stiffness and hyperuricemia in childhood CKD. To our knowledge, this study is the first to reveal the association between uric acid level and arterial stiffness in children with early-stage CKD. However, we found that cIMT was not elevated in children with CKD stage G2–G3. It is possible that arterial stiffness enables evaluation of arterial dysfunction, which may precede structural vascular remodeling evaluated by cIMT. Nevertheless, our cross-sectional study cannot determine whether increased arterial stiffness precedes the elevation of cIMT. Although PWV and AASI have been used in youths, age-specific reference ranges and reproducibility studies are still lacking [2]. Whether assessments of arterial-stiffness indices are superior to measure of vascular structure for predicting cardiovascular risk in children with early-stage CKD awaits further elucidation.

Recent evidence reveals that blood TMAO levels are elevated in adult patients with CKD, which is related to a poor long-term outcome [15]. However, little is known about the impact of urinary levels of TMAO-related metabolites in childhood CKD. A previous report demonstrated that the increases in blood TMAO in earlier CKD stages are driven by reductions in renal excretion [17]. This notion is supported by our results showing that children with CKD stage G2–G3 had a lower urinary level of DMA and TMAO than those with stage G1. Additionally, we found that urinary DMA level was negatively correlated with SBP. In normal subjects, urine TMAO displays a positive correlation with plasma TMAO [27]. However, urine TMAO was equivalent in adult CKD patients and controls [17]. Consequently, more studies are required to simultaneous determinations of TMAO-related metabolites in the blood and urine for their utility in predicting CVD, as well as the identification of TMA-producing bacteria in children with early-stage CKD.

In addition to structural and functional surrogate markers, in this study we examined microbial markers related to CV risk. We found that decreased abundance of the genus *Prevotella* was noted in CKD children with an abnormal ABPM profile, which was in agreement with a previous study showing that the genus *Prevotella* is associated with CVD risk [28]. According to our results, several beneficial microbes, such as *Lactobacillus, Bifidobacterium,* and *Akkermansia* [23,29], were not different between children with CKD stage G1 and G2–G3. However, the abundances of genera *Bifidobacterium* and *Lactobacillus* were correlated with urinary TMAO level. Additionally, the abundance of the genus *Lactobacillus* was negatively correlated with PWV. Therefore, some specific bacteria might serve as microbial markers related to CV risk in early-stage CKD youths. Conversely, our results contradict those previously published studies using the *Firmicutes* to *Bacteroidetes* ratio as a microbial marker for hypertension [9,10,11,12]. This is possibly because we were studying CKD children preceding hypertension onset but not in the stage of established hypertension. Interestingly, children with CAKUT had higher abundances of genera *Bifidobacterium* and *Lactobacillus* than those with non-CAKUT. Whether these alterations of beneficial microbes in the gut contribute to better renal survival and less CVD in patients with CAKUT than those with non-CAKUT deserves further evaluation [30,31].

This study has some limitations. First, there was no difference in cIMT among CKD youths. Larger numbers of patients might be needed to elucidate a true relationship. Second, our cross-sectional study cannot determine causality. Third, there might be an ethic difference as we used ABPM reference values from studies performed in Germany [21]. Next, we did not recruit normal healthy controls because we examined the difference of surrogate cardiovascular markers and gut-microbiota composition between two levels of renal function. That is, in the present study, children with CKD stage G1 served as the controls. Lastly, there are no reference values established for cIMT, PWV, AASI, and TMAO to define a cut-off point between healthy and CKD youths [2,15]. Additional studies for the normalization and validation of these surrogate markers for future application in daily practice are warranted.

## 5. Conclusions

In conclusion, BP abnormalities are enormously prevalent in children and adolescents with early-stage CKD. Although the mechanism underlying gut microbiota and CVD in CKD children has not yet been fully elucidated, our results have highlighted the link between gut microbiota, microbial metabolite TMAO, BP load, and arterial-stiffness indices in youths with early-stage CKD. Early detection of structural, functional, and microbial surrogate markers may assist in preventive care with respect to improving cardiovascular outcome in children with CKD.

## Figures and Tables

**Table 1:** Anthropometric and biomedical characteristics in children and adolescents with chronic kidney disease (CKD) stage G1–G3.

**CKD Stage**	**G1**	**G2–G3**
	*N* = 60	*N* = 26
Gender: M:F	35:25	19:7
Underlying disease: CAKUT: non-CAKUT	33:27	22:4 *
Age, years	9.5 (5.2–13)	13.7 (7.9–16.2) *
Body height, percentile	50 (25–75)	25 (15–75)
Body weight, percentile	50 (15–85)	25 (15–78)
Systolic blood pressure, mmHg	106 (99–115)	119 (108–127) *
Diastolic blood pressure, mmHg	68 (61–77)	70 (63–79)
Body mass index, kg·m^−2^	17 (15.6–20.8)	17.3 (15.7–23.5)
Hypertension (by office blood pressure)	18 (30%)	11 (42%)
Blood urea nitrogen, mg/dL	11 (10–13)	17 (13–20) *
Creatinine, mg/dL	0.48 (0.39–0.53)	0.89 (0.65–1.18) *
eGFR, mL·min^−1^·1.73 m^−2^	113 (103–129)	79 (54–85) *
Urine total protein-to-creatinine ratio, mg/g	64 (38–262)	57 (35–213)
Hemoglobin, g/dL	13.4 (12.8–14.1)	14 (12.5–15.4)
Total cholesterol, mg/dL	163 (142–197)	171 (147–182)
LDL, mg/dL	90 (68–111)	88 (77–100)
Triglyceride, mg/dL	67 (52–101)	70 (52–103)
Glucose, mg/dL	86 (82–91)	89 (85–93)
Uric acid, mg/dL	4.8 (3.9–5.7)	6.6 (5.9–7.6) *
Sodium, mEq/L	141 (140–142)	141 (140–142)
Potassium, mEq/L	4.4 (4.2–4.6)	4.4 (4.2–4.6)
Calcium, mg/dL	9.6 (9.2–9.9)	9.8 (9.5–10)
Phosphate, mg/dL	4.9 (4.7–5.3)	4.8 (4.1–5.1)

Data are medians [25th, 75th percentile] or n (%). * *p* < 0.05 by the Chi-square test or Mann–Whitney U-test. CKD: chronic kidney disease; CAKUT: congenital anomalies of the kidney and urinary tract; eGFR: estimated glomerular filtration rate; LDL: low-density lipoprotein.

**Table 2:** ABPM profile and cardiovascular assessment in children with CKD stage G1–G3.

**CKD Stage**	**G1**	**G2–G3**
24 h ABPM	*N* = 35	*N* = 19
Abnormal ABPM profile (with any of the following abnormalities) Average 24 h BP > 95th percentile Average daytime BP > 95th percentile Average nighttime > 95th percentile BP load ≥ 25% Nocturnal decrease of BP < 10%	20 (57%) 3 (9%) 3 (9%) 7 (20%) 14 (40%) 14 (40%)	15 (79%) 3 (16%) 4 (21%) 6 (32%) 14 (74%) * 8 (42%)
Cardiovascular assessment		
cIMT, mm	0.4 (0.3–0.4)	0.3 (0.3–0.4)
PWV, m/s	3.8 (3.4–4.2)	4.1 (3.7–4.9) *
AASI	0.36 (0.24–0.44)	0.38 (0.32–0.41)

Data are medians (25th, 75th percentile) or n (%). *p < 0.05 by the chi-square test or Mann–Whitney U-test. ABPM: 24 h ambulatory blood pressure monitoring; cIMT: carotid artery intima–media thickness; PWV: pulse wave velocity; AASI: ABPM-derived arterial-stiffness index.

**Table 3:** Urinary levels of DMA, TMA, and TMAO in children with CKD stage G1–G3.

**CKD Stage**	**G1**	**G2–G3**
	*N* = 60	*N* = 26
Urine level, ng/mg Cr		
DMA	234 (160–300)	177 (137–228) *
TMA	8 (5.5–14.8)	6.9 (3.8–11.2)
TMAO	344 (200–853)	209 (155–412) *

Data are medians (25th, 75th percentile). *p < 0.05 by the Mann–Whitney *U*-test. DMA: dimethylamine; TMA: trimethylamine; TMAO: trimethylamine N-oxide.

**Table 4:** Correlation between gut microbial composition and cardiovascular surrogate markers in children with CKD stage G1–G3.

**Abundance of Genus**	**cIMT**		**PWV**		**AASI**		**DMA**		**TMA**		**TMAO**	
	*r*	*p*	*r*	*p*	*r*	*p*	*r*	*p*	*r*	*p*	*r*	*p*
*Bacteroides*	−0.007	0.956	0.086	0.518	−0.052	0.709	0.072	0.508	−0.001	0.991	0.024	0.827
*Blautia*	−0.139	0.293	0.055	0.68	0.011	0.939	0.107	0.325	−0.05	0.646	0.307	0.004 *
*Faecalibacterium*	−0.234	0.074	0.014	0.916	−0.066	0.635	0.047	0.665	−0.079	0.468	0.072	0.508
*Bifidobacterium*	−0.01	0.94	−0.013	0.919	0.044	0.752	0.227	0.036 *	−0.025	0.817	0.36	0.001 *
*Oscillospira*	0.186	0.159	0.029	0.829	0.264	0.054	−0.038	0.725	0.111	0.311	−0.031	0.776
*Parabacteroides*	0.112	0.4	0.052	0.693	−0.003	0.981	0.053	0.625	−0.144	0.185	0.03	0.785
*Clostridium*	−0.011	0.936	0.196	0.137	0.044	0.75	−0.065	0.554	−0.038	0.728	0.13	0.232
*Ruminococcus*	0.227	0.084	0.107	0.418	0.266	0.052	0.002	0.985	0.034	0.757	0.094	0.388
*Akkermansia*	0.018	0.89	0.076	0.565	0.007	0.959	−0.097	0.372	−0.023	0.832	−0.071	0.519
*Collinsella*	−0.75	0.573	0.239	0.068	0.081	0.559	0.112	0.307	−0.128	0.24	0.254	0.018 *
*Lactobacillus*	−0.284	0.029 *	0.066	0.622	−0.151	0.274	0.32	0.003 *	0.145	0.181	0.428	<0.001 *

*p < 0.05 by Pearson’s correlation coefficient.
